# Maggot Therapy as a Part of a Holistic Approach in the Treatment of Multimorbid Patients with Chronic Ulcer

**DOI:** 10.3390/clinpract11020049

**Published:** 2021-06-02

**Authors:** Tobias Romeyke

**Affiliations:** 1 Medical Informatics and Technology, Institute for Management and Economics in Health Care, UMIT—University of Health Sciences, 6060 Hall in Tirol, Austria; tobias.romeyke@ext.umit.at; 2 Complementary and Individualized Patient Centred Medicine, Pain Therapy, Acute Hospital for Internal Medicine, Waldhausklinik, 86391 Deuringen, Germany

**Keywords:** wounds, ulcer, larva, *Lucilia sericata*, maggot therapy, debridement, multimorbid, holistic care

## Abstract

Patients with chronic wounds (leg ulcers, decubitus, and diabetic foot ulcers) suffer from marked restrictions in their quality of life and can often no longer adequately carry out their everyday tasks. The need for nursing and medical care increases when other illnesses and complaints are present at the same time. Qualified wound care and the treatment of comorbidities are therefore of particular importance. The treatment of this disease, which is increasing in number, requires a holistic, multimodal treatment approach which, in addition to professional wound care, also includes comorbidities in the treatment. This case study describes an old treatment method for refractory wounds, the so-called “maggot therapy”, and shows how this is integrated into a holistic, multimodal therapeutic approach.

## 1. Introduction

Patients with chronic wounds, such as leg ulcers, diabetic feet, or pressure ulcers, are considerably restricted in their quality of life [1,2]. Chronification also reduces the physical functionality of the patient—a withdrawal from the social environment, impairments in well-being, and depression are often described [3]. Severe forms of the disease can lead to sepsis, the loss of extremities, and even death. In Germany alone, the proportion of patients with chronic wounds is estimated at over 900,000, with the most common type of ulcer being venous leg ulcer [4]. The economic burden of different types of wounds is considerable worldwide [5,6,7].

In the anamnesis, diagnosis, and therapy, infection prophylaxis, odor, pain, and amputation reduction, as well as patient satisfaction, are central key factors. In addition to classic conventional medical measures for wound treatment, so-called biosurgery is experiencing a renaissance. The term biosurgery, maggot debridement therapy or larval therapy, describes the use of larvae (larvae of the fly species *Lucilia sericata*) to remove dead tissue and slough from wound surfaces. Even indigenous people used fly larvae to clean wounds that have failed to heal [8].

William Baer reported in case studies on the treatment of osteomyelitis with maggots during World War I. Between 1930 and 1940, maggot therapy was an established form of therapy in over 300 US clinics. It was widely practiced until World War II. In the 1940′s, penicillin and other antimicrobials become available, which led to a sharp decline in the use of maggot therapy. Ronald A. Sherman and Edward Pechter were great advocates of this form of therapy, supporting larval therapy and examining its mode of action in studies [8,9,10].

Nowadays, larval therapy is also increasingly used in response to the growing challenges posed by multiresistant bacteria that are often found in chronic wounds [11]. Maggot therapy is an evidence-based therapy method in medicine. The larval therapy is based on the action of proteases contained in the saliva of the larvae. This liquefies the dead tissue and then the larvae suck it up as food. An antibacterial peptide (lucifensin) and an antifungal peptide (lucimycin) have been detected in the larval secretions [12,13]. 

Digestive secretions released by the maggots promote wound healing and have an antibacterial effect [14]. The maggots are left on the wound as a rule for 48 h up to 4 days [15,16]. Pain can result as side effects of larval therapy [17], which is why an accompanying measurement using the visual analogue scale is necessary [18].

Maggot therapy is used for a wide range of chronic and acute wounds that require debridement and infection control [19,20,21,22]. In terms of holistic therapy, the physical limitations caused by the wound and the patient’s comorbidities should also be included in the treatment procedure [23]. Holistic therapy is understood to be an extension of methods in the field of medicine, which consider and refer to treating the whole person rather than individual symptoms in a comprehensive context. A therapy plan with therapy goals is drawn up based on the patient’s individual clinical picture, including the treatment of comorbidities. In addition to physiotherapeutic/physical, phytotherapeutic procedures, aspects of nutrition, and psychological well-being are emphasized. The consideration of somatic, therapeutic, and social aspects of the individual patient plays a special role.

The following case study describes a patient with several acutely exacerbated chronic wounds and a venous leg ulcer that was treated with maggot therapy. Due to the complex comorbidities with limited physical functionality, a holistic, complementary medical therapeutic approach was also used.

## 2. Case Presentation

### 2.1. Diagnosis

1. Ulcus cruris varicosum, left lower leg, with chronic venous insufficiency with signs of inflammation and therapy-resistant course, first diagnosis, 03/1994.
  Locations of the lesions:
  - Lower leg left medial, distal third (12 cm × 4.6 cm), in existence since 1994;
  - Lower leg left medial, proximal from 1. (2 cm × 1 cm), time of origin unknown;
  - Lower leg left medial, anterior from 1. (2.5 cm × 1.5 cm), time of origin unknown;
  - Lower leg left lateral, malleolus lateralis (5 cm × 3 cm), since the end of 05/2020;
  with sharp, tense pain.
2. Diabetic foot ulcer, left midfoot, no bacterial infection, since 1992;
3. Pressure point with hematoma and fresh lesion 0.5 cm in diameter, right dorsum pedis between MCT 3 and 4, distal third;
4. Lymphedema lower third of lower leg and left foot;
5. Benign paroxismal vertigo;
6. Normochromic normocytic anemia in chronic inflammation;
7. Status after thrombosis of the popliteal vein and all 3 lower leg veins on the left, first diagnosis 04/2019;
  - History of anticoagulation with Clexane.
8. Tendency to fall;
9. Urge incontinence;
10. Condition after hysterectomy and ovariectomy approx. 5 years ago;
11. Condition after appendectomy at the age of 9–10 years;
12. Condition after febrile bronchitis, 06/2019;

### 2.2. Cases Characteristics

In 1994, the patient’s medical history received diagnostics and therapy suggestions from several specialists regarding her chronic wound. Since then, the patient has avoided medical care and took care of the wound herself. In spring 2019, a thrombosis of the left popliteal vein was diagnosed. This was followed by a brief (03–04/2019) professional wound care in a wound center. The daughter states that this wound treatment worsened her wound status, so that the therapy in the wound center was discontinued and wound care was taken over again under the care of a general practitioner.

Up to this point, wound care had been carried out independently and with the help of the daughter at home every 3 days with Octenisept, calendula balm, sterile compresses, and a pressure bandage. After the last dressing change, the patient noticed “worms” in the wound (US left lat., Since 05/2020). This larvae infestation is called myiasis. In addition, the wound has enlarged since the previous dressing change and has become painful. Together with the daughter, the patient cleaned the wound and freed it from the parasites.

No current myiasis could be confirmed upon admission to hospital. In addition, there was a pronounced lymphedema on the left lower leg and foot. In addition, the patient complained about weakness and pain-related restricted mobility, especially on the left lower leg. She suffers from paroxysmal positional vertigo and back pain.

The family doctor refered the patient for further diagnostics, therapy, and professional wound care. Therapy with fly larvae (*Lucilia sericata*) was planned to improve the condition of the wound.

## 3. Diagnostic

Vegetative anamnesis: difficulty falling asleep and staying asleep, pain-related. No sweat, no fever. No alcohol, no nicotine. Appetite normal. No allergies. Urinary and stool continent. Height: 167 cm. Weight: 57 kg.

Head/neck: No pressure or tapping pain on the head. NAP free. Isokor pupils, light reaction consensually on the same side. Hearing inconspicuous. Moist oral mucosa, no fetus, tongue coated with white. Restored teeth. Posterior wall of throat not reddened.

Outer neck/thorax: Lymph nodes normal. No stridor. Thyroid normal, difficult to swallow. No jugular vein congestion. No pathological flow noise over the carotids. Chest normal. Axillary lymph nodes not enlarged. Breathing normal.

Pulmo: Sonorous knocking sound, vesicular breathing sound. No rattling noises.

Cor: Heart action regularly. HR 70/min. RR 12/mmHg.

Abdomen: Abdominal wall soft. No defensive tension. Liver and spleen not enlarged palpable. Kidney beds free on both sides. No resistance. No hernias. No lymph nodes. Gut noises downright. No pressure pains.

Spine: Pressure pain lumbar spine. No blockage. Mild kyphosis. No myogeloses. Lasegue negative. FBA 20 cm.

Cervical spine: Inclination/reclination: 30–0–30; Sidebend: 30–0–30; Rotation: 70–0–70.

Peripheral pulse status: ADP and ATP on both sides properly.

Neurology: Grossly neurologically normal. No pathological reflexes. Cranial nerves normal. No meningism. No tremor. The patient is psychologically clear, approachable, oriented on all sides.

Skin: Wound on both legs.

ECG upon admission: SR with HR 77/min, normal posture, no disturbance of arousal regression.

24 h ECG measurement: Assessment: Sinus rhythm with isolated SES, HR from a minimum of 58/min to a maximum of 116/min. Average heart rate 75/min. Two times a supraventricular tachycardia of 138–146/min. (longest 7 strokes). Isolated polytopic VES.

Nasopharyngeal swab for SARS-CoV-2 on admission: Negative.

## 4. Treatment and Progress

**Figure 1:**
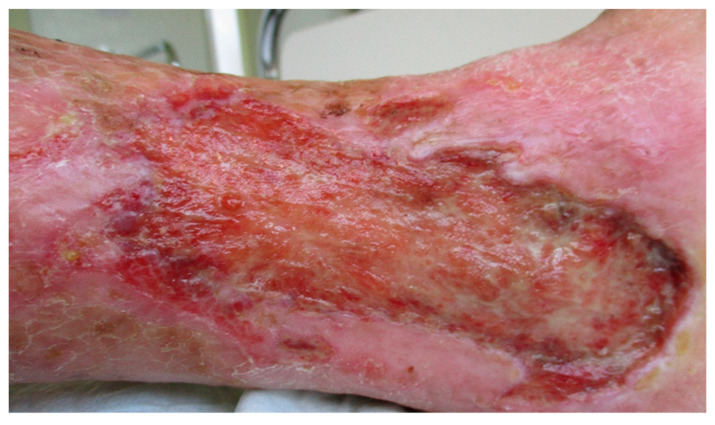
Venous leg ulcer before maggot therapy.

The 81-years-old patient was admitted to the hospital in a reduced general condition if the wound status deteriorates. In addition, there were weakness, reduced mobility, and sleep disorders. The patient withdrew more and more from society, and depression and anxiety often appeared (Figure 1).

The hospital stay was planned for maggot therapy with *Lucilia sericata* because of a therapy-resistant chronic venous leg ulcer, which had existed since 1994, as well as treatment of her comorbidities and complaints. Due to the wound care and the treatment of the companions, an inpatient stay with a multimodal holistic therapy of 16 days was obtained.

Before initiating maggot therapy, the hyperkeratosis was removed and both ulcers were debrided with a curette.

For chronic venous leg ulcers with existing signs of inflammation, fly larvae therapy was carried out with *Lucilia sericata* in a biobag, i.e., a complete polyester bag with larvae from BioMonde (Barsbüttel, Germany). In this case, we used BioBag^®^ 300: 1 polyester mesh bag (60 × 120 mm, with at least 300 living larvae of *Lucilia sericata* in the 1st and 2nd larval stage, as well as a BioBag^®^ 50: 1 polyester mesh bag (25 × 40 mm), with at least 50 living larvae *Lucilia sericata*.

Daily wound documentation was carried out by the wound managers according to the expert standard care of people with chronic wounds of the German network for quality development in care (DNQP). In the resorptive phase, the formation of granulation tissue was initially evident. On the fifth day, further expansion of the wound was found (fibroblasts form collagen). In the further course, scar tissue emerged (white-mother-of-pearl). It can be assumed that the repair phase had started as new epithelial cells had grown into the wound margins. The wound was then cleaned daily with Octenisept^®^ (anitiseptic), sterile bandages with HydroClean^®^ (interactive wound pillows with the suction–irrigation mechanism), and Zetuvit^®^ (absorbent compress), and a compression bandage was applied to both legs. A significant improvement in the wound conditions was achieved during the stay.

**Figure 2:**
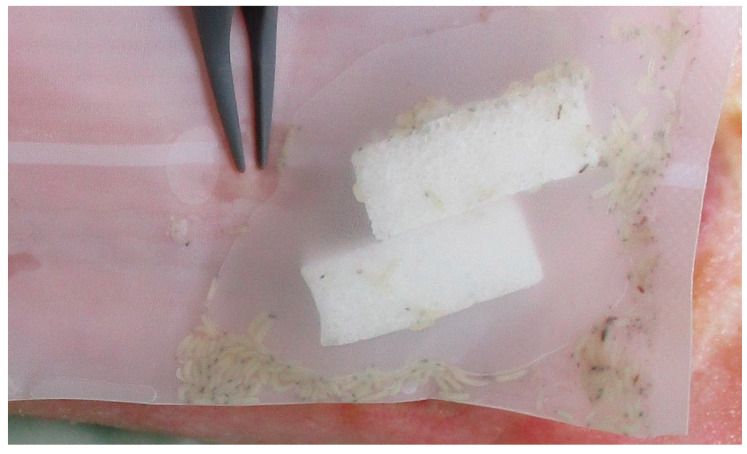
*Lucilia sericata* larvae enclosed in a biobag and applied to the wound.

**Figure 3:**
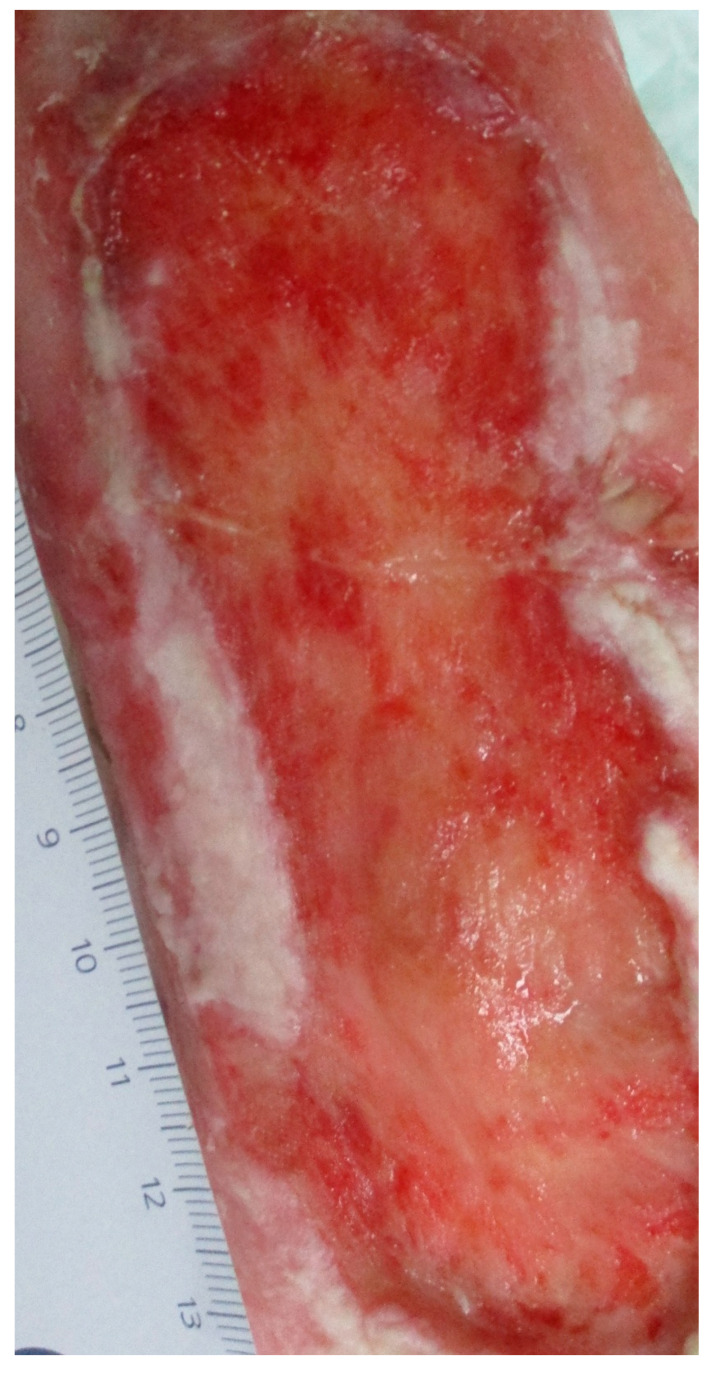

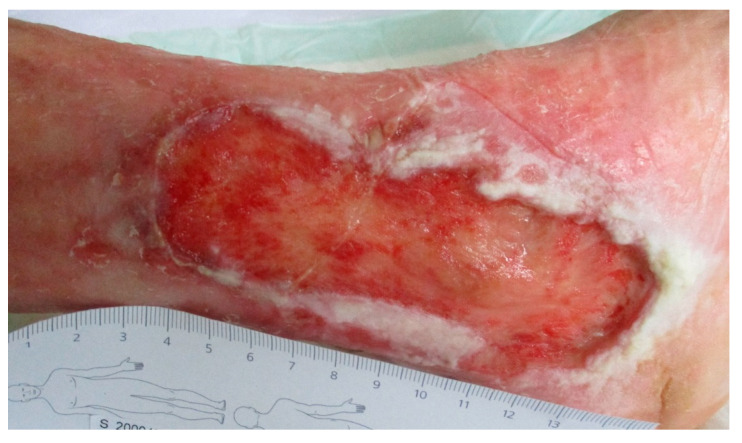
Ulcer post-larvae application (2 pictures of the same wound).

Figure 2 and Figure 3 show the progression of wound debridement during the study intervention.

The inflammation values showed a decrease in the course (Table 1). A normochromic, normocytic anemia most likely in the context of chronic inflammation was seen.

The patient was given protein-rich food to promote wound healing. In addition, extensive nutritional advice was given in this regard. Zinc and selenium were administered.

A long-term ECG was performed if the patient was dizzy. There was a sinus rhythm with a heart rate of a minimum of 58/min to a maximum of 116/min with a mean heart rate of 75/min on a daily average. Supraventricular tachycardia with a heart rate of 138–146/min (maximum 7 beats) was seen twice.

As part of the multimodal holistic therapeutic approach, high-frequency, and close-meshed procedures of phytotherapy, order therapy, exercise therapy, and acupuncture were also carried out.

The physiotherapeutic and physical interventions (massage therapy, physiotherapy, dizziness training, occupational therapy exercises) as well as naturopathic procedures to promote mobilization and pain reduction were well received by the patient and rated as positive.

With initial pain in the wound area, the patient received analgesic metamizole after a pain therapy prescription. This could be removed again during the stay.

In addition, the patient received acupuncture and intra-arterial therapeutic local anesthesia with 10 mL procaine 1% in the left femoral artery.

The patient also took part in psychotherapeutic group therapies, during which individual psychological and social coping strategies were developed. The patient was taught specific relaxation techniques, and mental methods for coping with illness.

**Figure 4:**
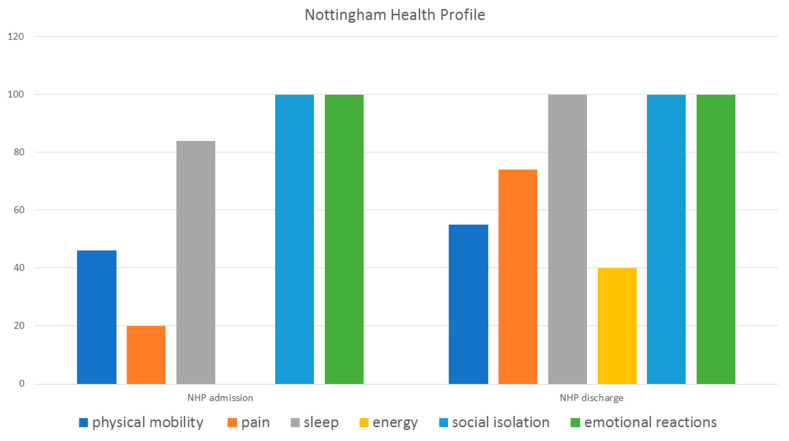
The quality of life on admission and discharge from the hospital measured with the Nottingham Health Profile (NHP).

In addition, interdisciplinary team meetings were held twice a week to evaluate the therapy goals. As a result of the high-frequency therapeutic approach, it was possible to improve the symptoms of the patient. In addition, the patient’s quality of life, measured with the Nottingham Health Profile (NHP), could be improved. (Figure 4).

In the diagnostic follow-up, a reduced inflammation value could be determined before discharge from the hospital (C-reactive protein (CPR), mg/L 23.3; Table 1). The physical functionality measured with the FFbH could be increased from 44 to 52%.

Follow-up examinations by the general practitioner showed no complications. A renewed presentation in the outpatient clinic was not necessary.

## 5. Discussion

**Figure 5:**
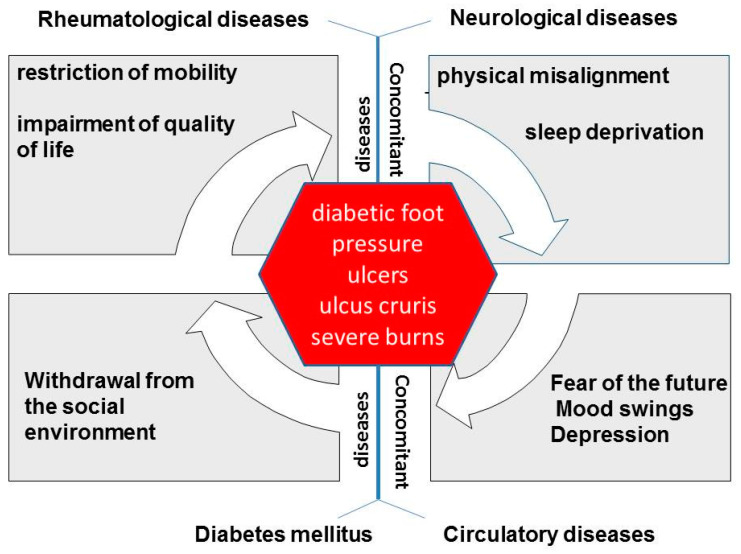
Vicious circle-wound and accompanying diseases.

The study shows a multimodal approach with the aim to break out of the vicious circled as visualized in Figure 5 wound and comorbidities.

Many patients with wounds that are difficult to heal also suffer from endocrinological, rheumatic, neurological, and/or cardiovascular diseases. In addition to increasing physical limitations, sleep disorders, depression, increasing withdrawal from social life, and even immobility can be the result [24,25].

Maggot therapy was integrated as an innovative therapy option for chronic wounds that are difficult to heal. It is also used for postoperative wound healing disorders, severe burns, osteomyelitis, or diabetic foot [26,27,28]. The positive wound cleaning effect of larval therapy has been proven in randomized, controlled clinical studies [16,29]. Three main therapeutic effects can be summarized: debridement, infection control, and stimulation of wound healing.

In addition to the therapy-resistant wound healing disorder, the patient had a chronic pain syndrome, increasing mobility restrictions, and a significantly reduced quality of life. As a part of a holistic approach, the integration of complementary therapeutic measures takes place. The comorbidities that exist in addition to the wound healing disorder are included in the treatment. The treatment aims to improve the patient’s quality of life.

One focus of the holistic approach is the integration of physical and physiotherapeutic measures [30]. The aim of these therapies was to increase the functional capacity and maintain and improve mobility through stretching and mobilization exercises since the patients are severely restricted in their mobility due to the pain in their extremities.

Pain can develop in and around the wound during and after therapy [31,32]. During the maggot therapy, the patient reported little or no pain in our case description (VAS 1–2/10). Fears that patients may develop during maggot therapy have not been described [31]. Overall, the comprehensive therapeutic approach resulted in a significantly improved quality of life.

Studies show also that the costs for wound treatment with maggot treatment are lower than with conventional wound therapy [33]. It is important, however, that maggot therapy is carried out by qualified wound managers so that the side effects mentioned are avoided.

## 6. Conclusions

Timely wound care with the use of maggot therapy, including the treatment of comorbidities, can increase physical functionality and additionally improve the quality of life of the patient. This is offered by a few clinics in Germany that specialize in the holistic treatment of patients. These treatment concepts include nutritional concepts to promote wound healing, exercise therapy, practicing therapy methods to regain mobility, psychotherapy to cope with illness, pain therapy methods to relieve pain and holistic care measures to improve quality of life [23].

## Figures and Tables

**Table 1:** Labor findings.

Indicator	Value	Further Information/Associations with Multiple Comorbidities
Neutrophil, Tsd/µL	5.84	Norm: 1.9–6.1 Infections with bacteria, viruses, fungi, or parasites can increase the value.
Basophil, Tsd/µL	0.02	Norm: ≤0.08. Diseases with higher concentrations of lipids in the blood (Diabetes mellitus, Nephropathien, Myxedema) can be associated with higher levels of basophiles.
Creatine, mg/dL	0.55	Norm: ≤1.10 (for individuals over 60). In case the norm is surpassed, the reasons are either acute kidney failure, chronic kidney disease, or desiccosis (lack of water, dehydration).
Lymphocyte absolute Tsd/µL	1.93	
C-reactive protein (CPR), mg/L	37.1	Norm: ≤5. Elevated CRP-levels are associated with bacterial and viral infections, rheumatic diseases, coronary diseases, heart attacks, etc.
Triglyceride, mg/dL	61	Norm: <150. Elevated values indicate metabolism disorders. Patients with diabetes, kidney diseases, or overweight often have higher levels.
Urea, mg/dL	26	Norm:10–50. Higher values in the blood serum indicate a reduced kidney function.
Uric acid, mg/dL	3.8	Norm: ≤7. Higher levels indicate chronic kidney diseases, diabetes, lipid metabolism disorders.
Lactate dehydrogenase, U/L	159	Norm: ≤250. Higher values indicate coronary heart diseases, myocarditis, cardiac arrhythmias, skeletal muscle diseases.
TSH basal µIU/mL	0.73	Norm: 0.27–4.2.
Protein electrophoresis		Albumin, Alpha-1-Globulin, Alpha-2-Globulin, Beta-Globulin, Gamma globulin. Indicates chronic liver diseases, acute and chronic inflammations, lack of protein, monoclonal gammopathies.
Albumin %	46.8%	Norm: 54.7–66.0
Alpha-1-Globulin %	7.2%	Norm: 3.1–5.6
Alpha-2-Globulin %	12.7%	Norm: 6.8–13.7
Beta-Globulin %	14.7%	Norm: 9.0–13.7
Gamma globulin %	18.6%	Norm: 10.6–19.8
Bilirubin mg/dL	0.21	Norm: 0–1.2 Higher values indicate damage to liver cells from drugs or infections with salmonella.
Erythrocytes Mio/µL	3.76	Norm: 3.9–5.2 If erythrocytes <3.9 that indicates anemia.
Iron µg/dl	22	Norm: 40–160 An iron deficiency leads to fatigue general feeling of weakness combined with fatigue and loss of performance, dizziness, and shortness of breath.
Hemoglobin g/dL	10.4	Norm: 12–16 A low hemoglobin level indicates that a person has too little iron in their blood. The transport of oxygen and the formation of new red blood cells (erythrocytes) are then at risk.
Hematocrit %	33	Norm: 36–46 A hematocrit value below the normal range can be the result of anemia or blood loss, but it can also be caused by excessive fluid consumption.
Red Blood Cell Distribution Width %	16.7	Norm: <15 Higher values indicate anemias (e.g., hemolytic anemia, iron deficiency anemia, pernicious anemia, or spheroidal cell anemia)

## Data Availability

Data is contained within the article.
